# Nanopore sequencing of infectious fluid is a promising supplement for gold-standard culture in real-world clinical scenario

**DOI:** 10.3389/fcimb.2024.1330788

**Published:** 2024-01-30

**Authors:** Manna Zhao, Yongyang Zhang, Li Chen, Xuebing Yan, Tianmin Xu, Maoying Fu, Yangguang Han, Ying Zhang, Bin Zhang, Juan Cao, Jing Lin, Dan Shen, Shuo Li, Chuanlong Zhu, Weifeng Zhao

**Affiliations:** ^1^ Department of Infectious Diseases, The First Affiliated Hospital of Soochow University, Suzhou, Jiangsu, China; ^2^ Department of Infectious Diseases, Nanjing Drum Tower Hospital, Affiliated Hospital of Medical School, Nanjing University, Nanjing, Jiangsu, China; ^3^ Department of Infectious Disease, The Affiliated Hospital of Xuzhou Medical University, Xuzhou, Jiangsu, China; ^4^ Department of Infectious Diseases, The Third People’s Hospital of Changzhou, Changzhou, Jiangsu, China; ^5^ Infectious Diseases Department, Kunshan First People’s Hospital, Kunshan, Jiangsu, China; ^6^ Department of Infectious Diseases, The Affiliated People’s Hospital of Jiangsu University, Zhenjiang, Jiangsu, China; ^7^ Department of Infection Medicine, The Fifth People’s Hospital of Wuxi, Affiliated Wuxi Fifth Hospital of Jiangnan University, Wuxi, Jiangsu, China; ^8^ Department of Infectious Diseases, Affiliated Hospital of Nantong University, Nantong, Jiangsu, China; ^9^ Emergency Department, Shanghai Shibei Hospital, Shanghai, China; ^10^ Key Laboratory of Digital Technology in Medical Diagnostics of Zhejiang Province, Dian Diagnostics Group Co., Ltd., Hangzhou, Zhejiang, China; ^11^ Medical Department, Nanjing Dian Diagnostics Group Co., Ltd., Nanjing, Jiangsu, China; ^12^ Department of Infectious Diseases, The First Affiliated Hospital of Nanjing Medical University, Nanjing, Jiangsu, China

**Keywords:** metagenomic next-generation sequencing (mNGS), diagnosis, infectious diseases, infectious body fluids, nanopore sequencing technology (NST)

## Abstract

**Introduction:**

Infectious diseases are major causes of morbidity and mortality worldwide, necessitating the rapid identification and accurate diagnosis of pathogens. While unbiased metagenomic next-generation sequencing (mNGS) has been extensively utilized in clinical pathogen identification and scientific microbiome detection, there is limited research about the application of nanopore platform-based mNGS in the diagnostic performance of various infectious fluid samples.

**Methods:**

In this study, we collected 297 suspected infectious fluids from 10 clinical centers and detected them with conventional microbiology culture and nanopore platform–based mNGS. The objective was to assess detective and diagnostic performance of nanopore-sequencing technology (NST) in real-world scenarios.

**Results:**

Combined with gold-standard culture and clinical adjudication, nanopore sequencing demonstrated nearly 100% positive predictive agreements in microbial-colonized sites, such as the respiratory and urinary tracts. For samples collected from initially sterile body sites, the detected microorganisms were highly suspected pathogens, and the negative predictive agreements were relatively higher than those in the microbial-colonized sites, particularly with 100% in abscess and 95.7% in cerebrospinal fluid. Furthermore, consistent performance was also observed in the identification of antimicrobial resistance genes and drug susceptibility testing of pathogenic strains of *Escherichia coli*, *Staphylococcus aureus*, and *Acinetobacter baumannii*.

**Discussion:**

Rapid NST is a promising clinical tool to supplement gold-standard culture, and it has the potential improve patient prognosis and facilitate clinical treatment of infectious diseases.

## 1 Introduction

Infectious diseases are significant causes of morbidity and mortality worldwide as well as spreading rapidly (Allerberger and Kern, 2020), with the complicated and multi-sites of occurrence. The annual incidences of meningitis, sepsis, and respiratory tract infections (RTIs) are approximately 850,000, 5.68 million, and 10 million in China, with a high mortality rate caused by confusing identification of the pathogen, complex etiology, and clinical decision challenges (Bodilsen et al., 2021; Wu et al., 2021). Therefore, rapid identification and appropriate diagnosis of pathogens in infectious disease management are of great clinical importance.

Currently, traditional detection of pathogenic microorganisms mainly utilizes morphology and molecular detection based on culture, which are basic tools and gold standard for clinical results confirming (Pliakos et al., 2018), especially the detection of bacteria and fungi. However, culture-based pathogen identification also has shortcomings, such as low detection accuracy (Parize et al., 2017), long culture cycle, high requirements on personnel operation, and inevitable deviations in the culture of unculturable microbes (Srivastava and Prasad, 2023). Thus, these methods cannot meet the current needs for rapid and accurate infection diagnosis; as a result, metagenomic next-generation sequencing (mNGS) has emerged as a clinical complement for gold-standard culture or even more clinically needed.

Rapid mNGS testing is a culture-independent, rapid detection of unknown infections for body fluids. It mainly includes short-read sequencing represented by Illumina platform and long-read sequencing represented by Nanopore platform (Mitchell and Simner, 2019; Zhang et al., 2022). Early studies have shown that mNGS can reliably detect and identify causative or emerging microorganisms in unexplained infectious disease syndromes (Li et al., 2019; Diao et al., 2022) and facilitate adjustment of empiric antibiotic therapy (Feng et al., 2023). The Illumina sequencing platform has been widely used clinically, but its short read length limits applicability to genomes. With long-read sequencing, nanopore-sequencing technology (NST) exhibits potential to compensate for this and meet the clinical needs for pathogen detection in high-viral-load clinical samples (Li et al., 2023) and species identification of *Mycobacterium tuberculosis* isolates (Smith et al., 2020). Therefore, NST may be a promising complement for sequencing platforms currently used in clinical settings. However, few studies have reported the diagnostic presentation of mNGS at different sites of infection, especially based on the nanopore platform.

Here, we designed a multi-center, observational study to investigate the detective and diagnostic performance of NST based on the analysis of 297 samples of suspected infections. With the inclusion of seven types of clinical fluid samples, we aimed to provide supporting evidence to evaluate the clinical impact of NST on real-world infection samples.

## 2 Methods

### 2.1 Study design and participants

This study was a multi-center observational study, recruiting patients with suspected infections of respiratory, urinary, central nervous system, bloodstream, thoracic, abdominal, or local body sites who met the inclusion criteria (**Supplementary Table S1**). Study protocols were reviewed and approved by the Ethics Committee of The First Affiliated Hospital of Soochow University (No. 2021293) and filed by the Ethics Committees of the other nine clinical centers. Consent has been obtained from each patient after a full explanation of the purpose and nature of all procedures used.

### 2.2 Sample collection and conventional microbiological culture

Suspected infectious fluid specimens, namely, plasma, bronchoalveolar lavage fluids (BALFs), sputum, cerebrospinal fluids (CSFs), abscess, serous cavity effusions (SCEs) and urine, were clinically obtained from patients within 48h of admission or disease onset. In addition, clinical data were collected and the 14-day follow-ups were conducted after sample collection.

All the fluid samples were collected in sterile tubes with the fewest volumes of 3.0 mL for each patient. Fresh samples were sent for bacterial and fungal cultures as routine clinical detection. For bacterial culture, blood agar plates, chocolate agar plates, and MacConkey agar were used and the inoculated plates were cultured with 5% CO_2_ under 35°C for 48h. For suspected Mycobacterium culture, samples were inoculated to Roche medium for 7 days. As for fungal culture, Sabouraud Dextrose agar was used for 7-day culture for positive colonies. Positive colonies were species identified and antimicrobial resistance (AMR) testified with Gram staining, oxidase, and biochemical identification by automatic microbial identification system.

### 2.3 Nanopore sequencing and data analysis

Sample preparation, sequencing, and data analysis under nanopore platform were conducted as previously published (Gu et al., 2021; Luo et al., 2023). Basically, plasma samples were centrifuged and the cell-free DNA of supernatant were extracted (Gu et al., 2021). Other fluid samples were DNA extracted with the pellet (Luo et al., 2023). To reduce the high contamination of human genomes, effective host DNA depletion was applied (Charalampous et al., 2019), and the conducted protocol was as previously reported (Nelson et al., 2019). After host DNA depletion and microbial DNA extraction, the concentration of each sample was calibrated, followed by fragmentation, barcode ligation, library purification, and PCR amplification, according to manufacturer’s instructions (Oxford Nanopore Technologies, Oxford, UK).

After library preparation, sequencing was performed with the GridION platform (Oxford Nanopore Technologies, Oxford, UK) as previously described (Luo et al., 2023). Raw data of NST were generated with real-time identification by MinKnow software. After removing host DNA by Minimap2 Software, filtered data were multi-sequence aligned and standard as 20 million (M) each sample. The filtered data were identified with microorganism sequences at the National Center for Biotechnology Information (https://www.ncbi.nlm.nih.gov/). Considering the original blast results would contain redundant micro-ecosystem organisms, the reported criteria of pathogen were established. Due to the wide variety of sample types, samples collected from microbial-colonized body sites, such as BALF, sputum, and urine, the reported criteria were referenced as previously published (Luo et al., 2023). For samples of CSF, SCE, abscess, and plasma, the detected organisms would be reported as much as possible, after removing the procedure-background microbes and those sequences lower than three (except tuberculosis).

### 2.4 Statistics

The R software (version 4.1.0) was used for data process and result visualization (packages of mixOmics, ggplot2, and pheatmap). The chi-square test was applied to access the accuracy evaluation and diagnostic performance of mNGS, reported as sensitivity, specificity, positive predictive agreement, negative predictive agreement (Gu et al., 2021), positive predictive value, and negative predictive value with their 95% confidence intervals. *P* < 0.05 were considered with statistical significance.

## 3 Results

### 3.1 Study design and general characteristics of enrolled cohorts

**Figure 1:**
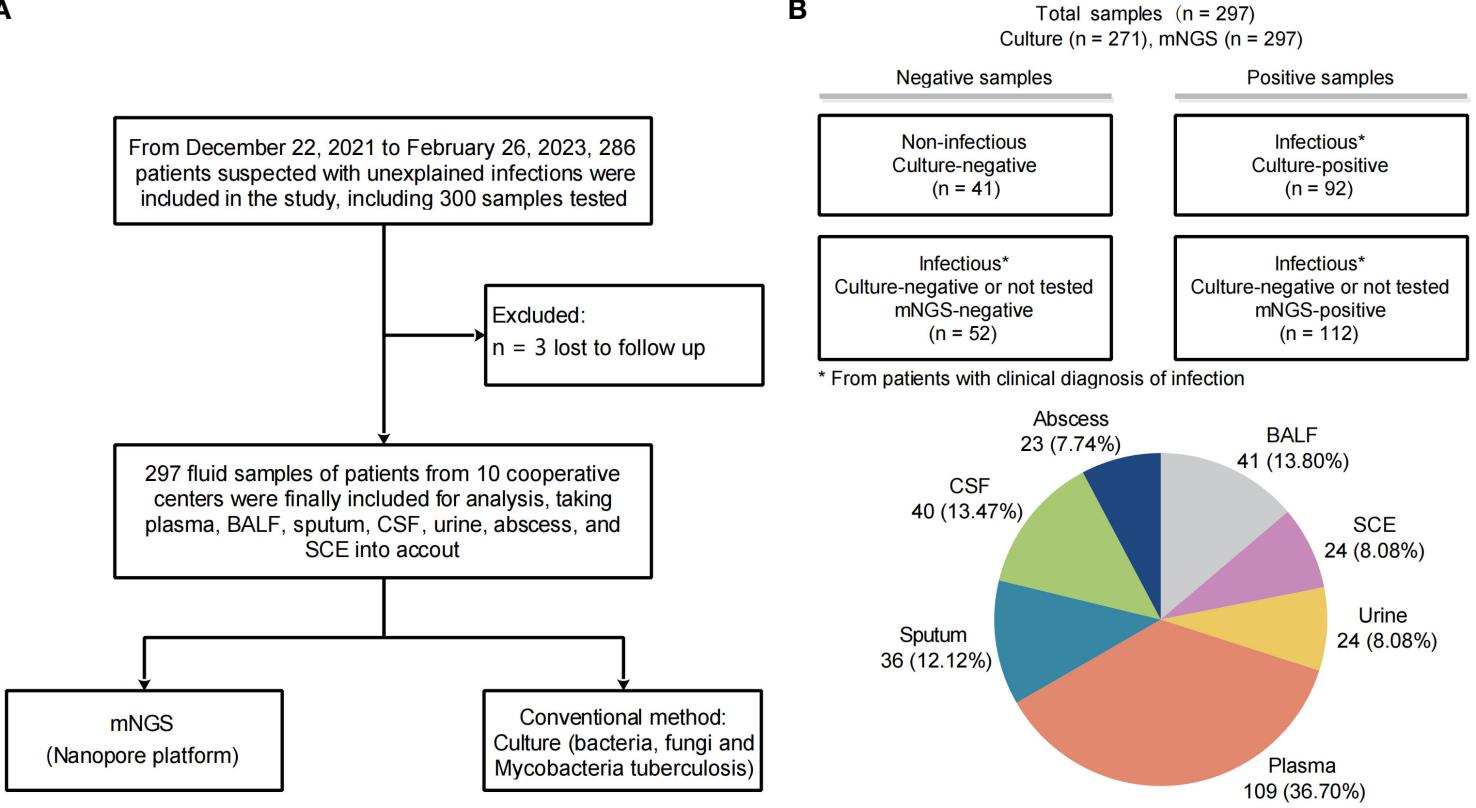
Study workflow and sample distribution. **(A)** Overview of patient enrollment workflow. **(B)** Analysis workflow and primary results of 297 fluid samples collected. The pie chart displays the fluid sample types analyzed in this study (*n*, %). *From patients with clinical diagnosis of infection.

A total of 297 samples from 286 patients were finally enrolled and analyzed for this study (**Figure 1A**). Baseline characteristics of patients and samples were listed in **Table 1** and **Supplementary Table S2**. The pathogen profiles were explored and evaluated using NST, and 271 of 297 were clinically conducted with conventional culture, as gold standard approach (**Table 2**). Based on our results, 256 samples clinically determined with infectious disease, 112 were only identified with NST, and 52 failed to be identified with neither culture nor NST (**Figure 1B**). Basic sequencing results of average length were presented in **Supplementary Figure S1A**. The length of sequencing reads of samples collected from microbial-colonized body sites was significantly longer than those of sterile sites.

**Table 1:** Enrolled patient characteristics.

Patient demographics (*n* = 286)	Value
**Sex, n (%)**	
Female	124 (43.3%)
Male	162 (56.7%)
**Age/years, mean (SD)**	54.8 (18.0)
**Body temperature/℃, mean (SD)**	38.1 (1.17)
**CRP^1^ ≥ 4.0 mg/L, n (%)**	207 (73.4%)
**PCT^2^ ≥ 0.5 ng/mL, n (%)**	89 (31.1%)
**Presumed illness, n (%)**	
Respiratory infection	90 (30.3%)
Bloodstream infection	36 (12.1%)
Central nervous system infection	24 (8.1%)
Local infection	31 (10.4%)
Urinary tract infection	19 (6.4%)
Multi-site infection	38 (12.8%)
Other infection	37 (12.5%)
Noninfectious	22 (7.4%)
**No empiric treatment while collection, n (%)**	83 (27.9%)
**14-day outcome, n (%)**	
Clinical cure	17 (5.7%)
Improvement	223 (75.1%)
No improvement	49 (16.5%)
Death	2 (0.7%)
Unclear	6 (2.0%)

^1^CRP, C-reactive protein.

^2^PCT, Procalcitonin.

### 3.2 Microbial identification landscape of mNGS test based on NST

After calculating the numbers of positive and clinically consistent tests, our results showed that the overall positive rates based on NST versus (vs.) clinic culture assay were 67.34% versus 33.95%, and clinical consistency were 67.34% versus 44.65%, respectively (**Table 2**). As for different types of samples, it could be recognized that NST performed better detection capability (higher positive detect rate) in almost all types of samples, especially for BALFs and sputum (about two times better than culture, both positive rate and clinical consistency). Those results also demonstrated that NST be capable to detect more varieties of microbes in real-world clinical samples.

**Table 2:** Comparison of positive rate and clinical consistency between culture and NST.

Sample type	Total test (n)		Positive rate (n, %)		Clinical consistency^1^ (n, %)	
	Culture	NST	Culture	NST	Culture	NST
BALF	34	40	12 (35.29)	35 (87.50)	10 (29.41)	31 (78.05)
Sputum	32	36	16 (50.00)	34 (94.44)	14 (43.75)	32 (88.89)
Urine	24	24	13 (54.17)	23 (95.83)	13 (54.17)	17 (70.83)
Abscess	21	24	12 (57.14)	22 (91.60)	13 (61.90)	23 (95.65)
Plasma	102	109	30 (29.41)	62 (56.88)	46 (45.10)	61 (55.96)
CSF	38	40	5 (13.16)	16 (40.00)	18 (47.37)	28 (70.00)
SCE	20	24	4 (20.00)	8 (33.33)	7 (35.00)	8 (33.33)
Total	271	297	92 (33.95)	200 (67.34)	121 (44.65)	200 (67.34)

^1^Clinical consistency was determined by the clinician, according to the laboratory tests, follow-up status, and treatment outcomes of enrolled patients.

**Figure 2:**
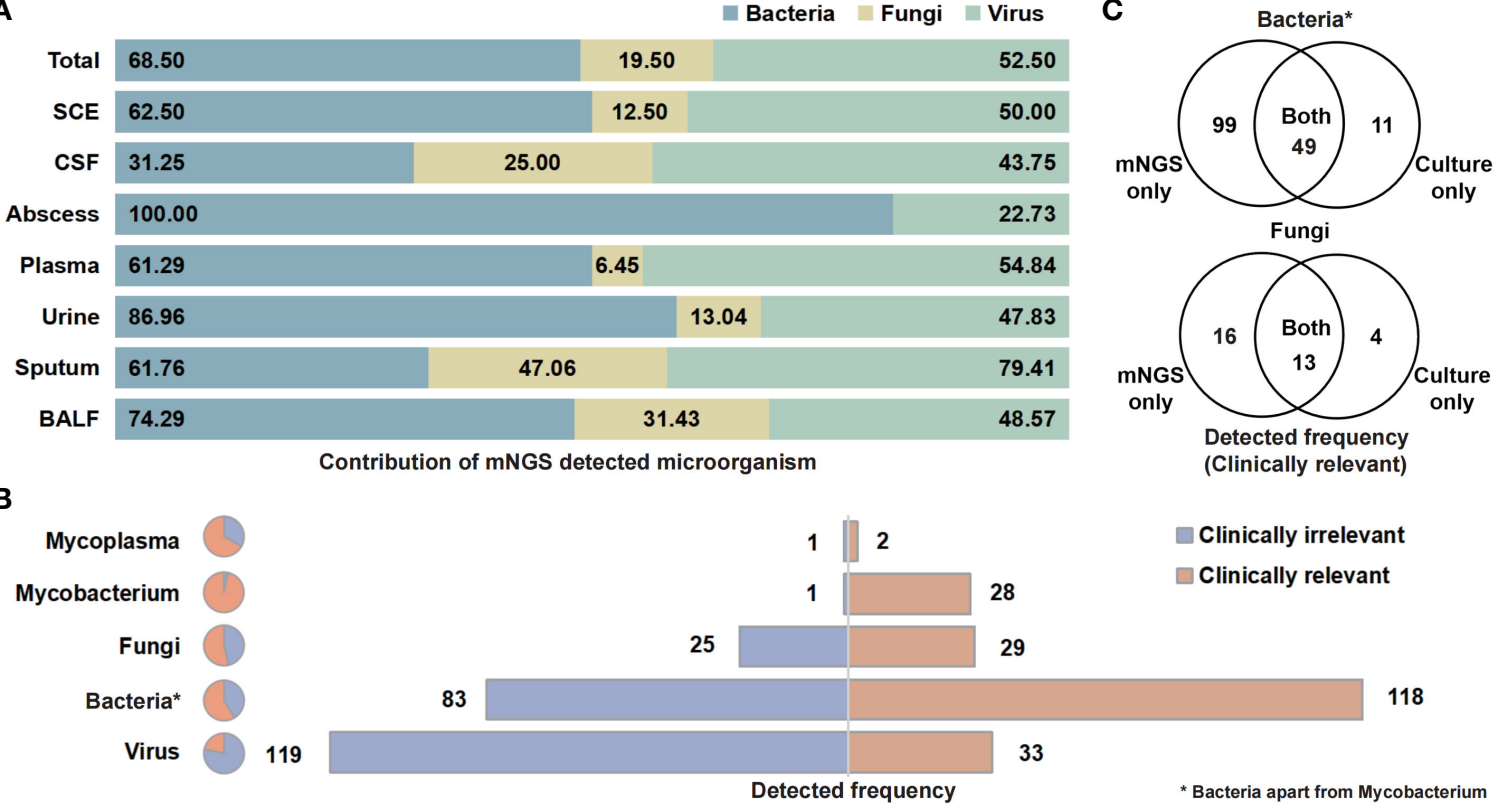
Contribution of NST identifying microorganism and the clinical relevance of taxonomic categories. **(A)** Percentage of NST-identified bacteria, fungi, and virus among fluid sample types. **(B)** Times of NST detection of taxonomic groups and the contribution of clinical relevance. **(C)** Test consistency of clinically relevant-bacteria and fungi between NST and culture. *Bacteria apart from *Mycobacterium*.

The categories and percentages of detected microorganisms were exhibited as **Figure 2A**. It should be noted that, because of the coexistence of numerous categories, the sum of percentages was over 100, and total detected frequencies were larger than number of samples. As for clinical meaning, 58.7% (118 of 201) bacterial (apart from *Mycobacterium*), 53.7% (29 of 54) fungal, and only 21.7% (33 of 152) viral detection were clinically relevant and determined as pathogens (**Figure 2B**). Among those clinically relevant microbes, 66.9% (99/148) bacteria (apart from *Mycobacterium*) and 55.2% (16 of 29) fungi could only be detected with NST (**Figure 2C**). For *Mycobacterium*, 96.6% (28 of 29) were detected as pathogen, while only one positive culture was observed in this study (**Supplementary Figure S2**).

### 3.3 Microbiome-dominant distribution of microbial-colonized body sites

To further evaluate the microbial distributing features of each type of sample, detailed taxonomic analysis was conducted in this study. For samples collected from microbial-colonized sites, 28 of 42 detected genus were with less than 50% clinical relevance, especially those of virus (only eight of 88, **Supplementary Figure S3A**). It enlightened us that clinical determinations and decision of mNGS-detected microorganisms are of vital importance for microbial-colonized sites.

**Figure 3:**
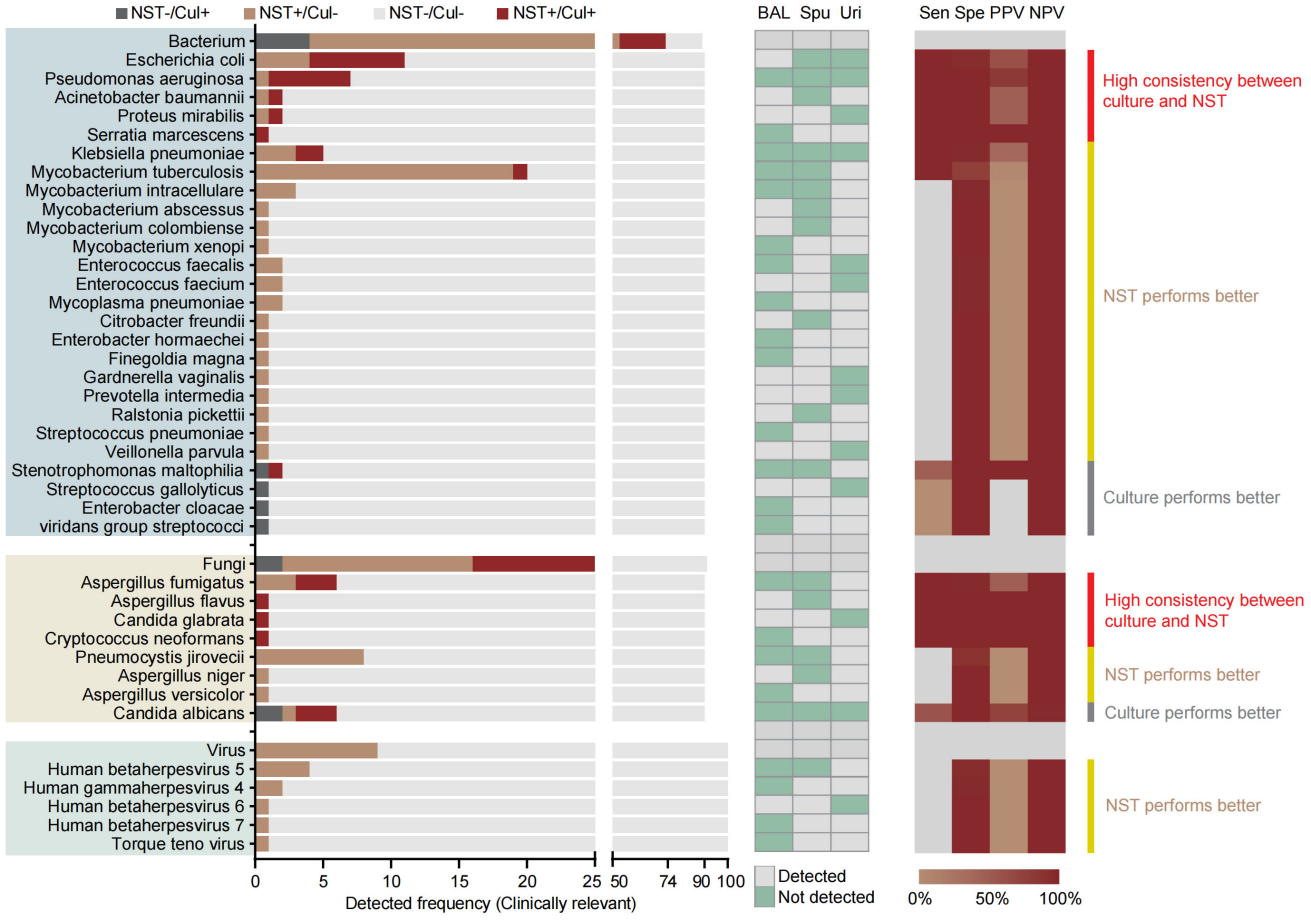
Distribution and statistic performance of clinically relevant pathogens identified with NST and culture (species level) from body sites with colonized microbiome, including BALF, sputum, and urine; Cul, culture; BAL, bronchoalveolar lavage fluid; Spu, sputum; Uri, urine; Sen, sensitivity; Spe, specificity; PPV, positive-predictive value; NPV, negative-predictive value.

As for species-level analysis, the clinically relevant microbes were selected here to calculate the diagnosis performance based on the NST (**Figure 3**). In total, 90 samples of microbial-colonized sites were tested by both NST and clinic culture. Our results showed that a high consistency of these two approaches for detection of *Escherichia coli* and *Pseudomonas aeruginosa* (sensitivity = 100% of 100%, specificity = 95% of 99%, respectively). On the other hand, for some pathogens, such as *Mycobacterium* spp., there was only one *Mycobacterium tuburculosis* (MTB) positively detected by clinic culture assay, while 19 MTB and 6 non-tuberculosis mycobacterium (NTM) were additionally detected with NST, from culture-negative samples. Moreover, the number of detective reads of top 10 clinically relevant pathogen was exhibited in **Supplementary Figure S1B**.

### 3.4 Pathogen-dominant identification of sterile body fluids

For aseptic body sites, results also give us insights that the positive detection of microbes could be more highly suspected pathogens, compared with microbial-colonized sites. The clinical relevance of detected bacteria, fungi and viruses were 84.0%, 60%, and 40%, respectively (**Supplementary Figure S3B**).

**Figure 4:**
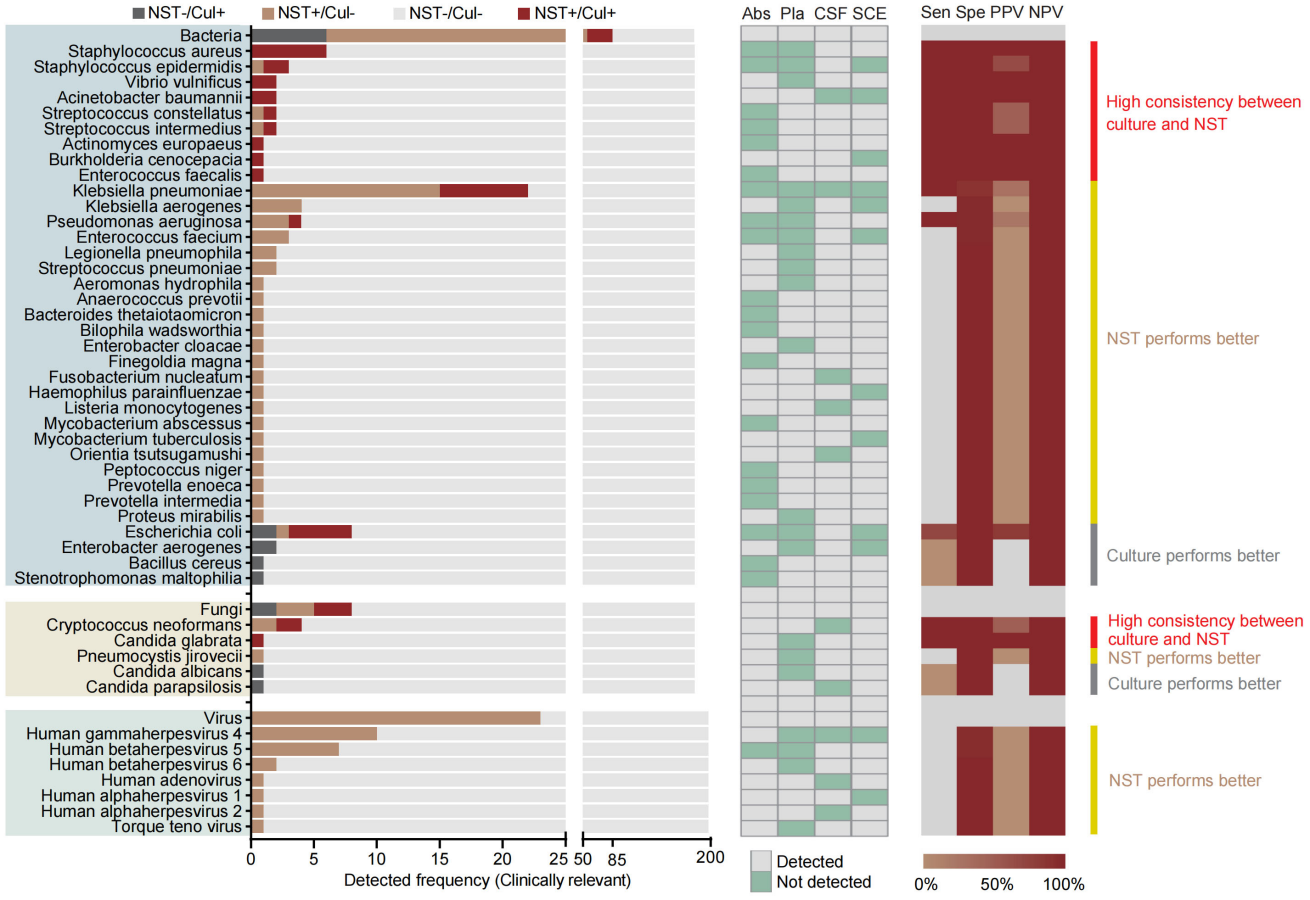
Distribution and statistic performance of clinically relevant pathogens identified with NST and culture (species level) from body sites with non-colonized microbiome, including abscess, plasma, CSF, and SCE. Cul, culture; Abs, abscess; Pla, plasma; CSF, cerebrospinal fluid; SCE, serous cavity effusion; Sen, sensitivity; Spe, specificity; PPV, positive predictive value; NPV, negative predictive value.

Species-level analysis was performed based on 181 samples from aseptic body sites, tested with both NST and clinic culture (**Figure 4**). Our results showed that a total of 36 pathogenic bacteria and five clinically relevant fungi were identified in 51 samples. Meanwhile, 79 bacterial and six fungal pathogens were identified in 108 samples using NST. Similar to microbial-colonized sites, the culture-detected fungi were mainly *Candida* spp. and *Cryptococcus neoformans*. However, we found that the culture-obtainable bacterial patterns of sterile sites were different compared with microbial-colonized sites, with relatively higher detected frequencies of *Klebsiella pneumoniae* and *Staphylococcus* spp. (22 vs. 5 and 9 vs. 0, respectively) and lower frequencies of *Mycobacterium* spp. (2 vs. 26).

### 3.5 Test accuracy of nanopore sequencing

Accuracy evaluation of each type of sample mainly focused on the performance of NST relative to gold standard culture (**Supplementary Table S4**). Because of the high false-negative rate of clinical routine culture, the composite standard additional with clinical adjudication (Gu et al., 2021) was also applied (**Table 2**). While utilizing composite standard evaluation, total positive percentage agreement (PPA) and negative percentage agreement (NPA) of NST were 89.7% [95% confidence interval (CI) 84.1%–93.5%] and 69.0% (95% CI 59.5%–77.2%) according to our results.

As regard to each fluid type, the PPA of microbial-colonized sites was 100%, while the NPA was relatively lower than the average level (14.3%–50.0% vs. 69.0%). It is supposed to be the presence of colonizers causing the difficulty of true-negative detection. For sterile body sites, performances in abscess and CSF showed both high PPA (95.7% and 88.2%) and high NPA (100% and 95.7%), suggesting a high confidence level for local and neurological infections. As for plasma samples, the PPA and NPA were 77.6% (95% CI 64.4%–87.1%) and 66.7% (95% CI 52.0%–78.9%). Interestingly, our results found that the statistical performances of SCE samples exhibited a relatively volatile 95% CI using NST.

### 3.6 Concordant performance of antimicrobial resistance gene identification and susceptibility test

**Figure 5:**
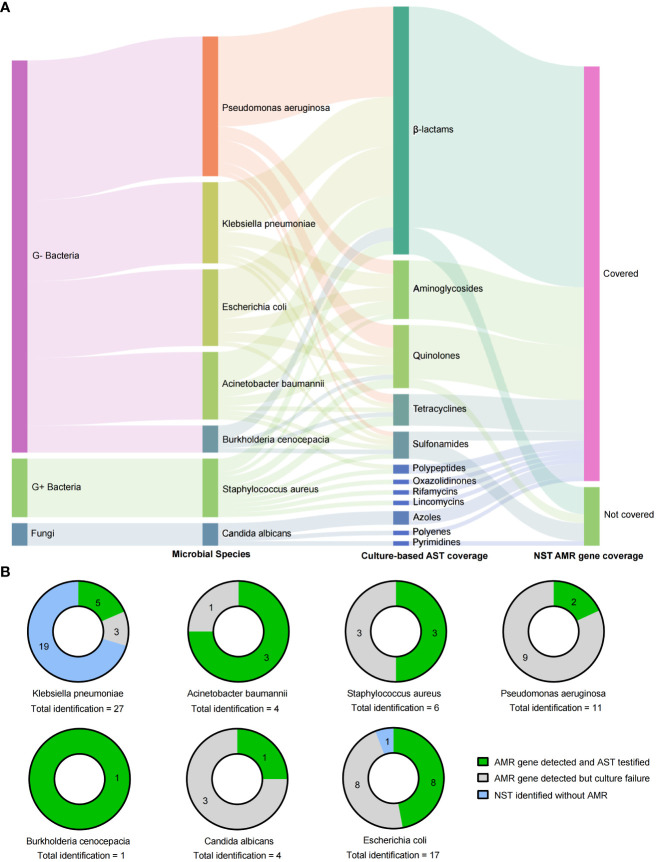
Drug resistance identification of clinically relevant pathogens with culture-based AST and NST-based AMR gene identification. **(A)** Summary of coverages of AST and AMR gene identification. **(B)** Clinically relevant Species identified with AMR genes and the concordance with AST; AMR, antimicrobial resistance; AST, antimicrobial susceptibility testing; G+/G−, Gram positive/negative.

In addition to the microbial identification, the AMR genotype information is also capable to explore by NST. In this study, there were 62 NST-detected microbes carrying with AMR genes, while only 25 of them with the positive cultural colonies for antimicrobial susceptibility testing (AST), as shown in **Figure 5**, and the antibiotics coverage of NST were shown as **Figure 5A**.

By comparing the consistency of AMR genotype and drug-resistant phenotype in two different approaches, we found that strains of *E. coli* were most frequently detected with AMR genes (16 of 17, with NST), and eight of them could be successfully cultured and testified with AST. Although strains of *Klebsiella pneumoniae* showed the most identification frequency (*n* = 27), the AMR-positive rate was eight of 27, and 62.5% (five of eight) of them could be testified with AST (**Figure 5B**). Those results indicated that nanopore sequencing could additionally provide supporting information to drug resistance identification.

## 4 Discussion

Fast and precise infection diagnosis makes a significant contribution to improving clinical management and patient prognosis, and mNGS is a promising tool to complement gold-standard culture in clinical practice. Although mNGS has been widely reported in the auxiliary diagnosis of infectious diseases, few studies have systematically evaluated the diagnostic performance of various fluid samples via NST in real-world infectious diseases, not to mention the comparison between sterile and microbial-colonized sites. This study included seven types of clinical fluids, from 10 clinical centers, and provided evidence to evaluate the effectiveness of nanopore sequencing in real-world clinical scenarios.

Nanopore sequencing exhibits fast detection (Gorzynski et al., 2022; Jain et al., 2022) and reduced turnaround time (Sheka et al., 2021) and enables both pathogen identification and AMR gene detection capabilities by unbiased rapidly sequencing. In addition, NST reduces the requirements of resource and technology, as well as capital and maintenance costs. Nanopore sequencing has been applied in genome assembly, full-length transcript detection, base modification detection, and other specialized areas, such as rapid clinical diagnoses and outbreak surveillance (Wang et al., 2021). The long-read length sequencing of NST could enhance the accuracy of real-time analysis of bioinformatics and, thus, provide opportunities for faster speed compared with other strategies using sequence-by-synthesis methods (Gu et al., 2019). In this regard, NST displays more potential for application as point-of-care testing (POCT) than Illumina platform (Ferreira et al., 2018); thus, the study on its true performance in clinical scenario could be of vital importance. Although mNGS is currently used for companion diagnosis, the diagnostic performances of NST in real-world clinical scenarios were rarely published. Most previous studies have only explored the diagnostic performance of mNGS with a single type or a limited number of samples, including bloodstream infections (sensitivity = 30.8%) (Hu et al., 2021), lower RTIs (sensitivity = 100%, specificity = 66%) (Yang et al., 2019) and meningitis (sensitivity = 41.7%, specificity = 78.6%) (Gao et al., 2021). In this study, the diagnostic performances in multiple types of samples were calculated based on a composite standard (**Table 3** and **Supplementary Table S4**). Even though the main disadvantages of mNGS, such as false positive and noise detection of background germs, were also observed in NST, efforts were made to discriminate positive detection with true pathogen by combined use of clinical adjudication in this study. This method has also been previously reported (Luo et al., 2023). The PPA and NPA of our results (**Table 3**) exhibited comparable or even better performances with the previous publishing mentioned above. In addition, numbers of the latest Chinese expert consensus recommended mNGS as supplemental detection of emerging pathogens and acute infections. It supports that nanopore-based mNGS has potential to be used as a promising supplement for the existing detection in clinical diagnostic routines.

**Table 3:** Statistical performance of different types of samples (*n* = 297, composite standard).

Sample type	TP	FP	TN	FN	PPA	NPA	PPV	NPV
BALF	30	5	5	0	100.0 (85.9–100)	50.0 (20.1–79.9)	85.7 (69.0–94.6)	100 (46.3–100)
Sputum	31	3	2	0	100.0 (86.3–100)	40.0 (7.3–83.0)	91.2 (75.2–97.7)	100 (19.8–100)
Urine	17	6	1	0	100.0 (77.1–100)	14.3 (0.8–58.0)	73.9 (51.3–88.9)	100 (5.5–100)
Abscess	22	0	1	1	95.7 (76.0–99.8)	100.0 (5.5–100)	100 (81.5–100)	50.0 (2.7–97.3)
Plasma	45	17	34	13	77.6 (64.4–87.1)	66.7 (52.0–78.9)	72.6 (59.6–82.8)	72.3 (57.1–83.9)
CSF	15	1	22	2	88.2 (62.3–97.9)	95.7 (76.0–99.8)	93.8 (67.7–99.7)	91.7 (71.5–98.5)
SCE	5	3	13	3	62.5 (25.9–89.8)	81.3 (53.7–95.0)	62.5 (25.9–89.8)	81.3 (53.7–95.0)
Total	165	35	78	19	89.7 (84.1–93.5)	69.0 (59.5–77.2)	82.5 (76.4–87.4)	80.4 (70.9–87.5)

TP, FP, FN, and TN were shown as numbers of tests. PPA, NPA, PPV, and NPV were shown as % (95% confidence intervals) and were calculated with http://vassarstats.net/clin1.html#return.

Considering that different types of samples characterize different sites of infection, we further analyzed the distributed features of body sites, including the microbial-colonized sites and originally sterile sites. A total of seven types of fluid samples were included in this study, three of them were collected from the respiratory and urinary tracts (BALF, sputum and urine), and the others were collected from sterile sites, where microbes could be detected only in pathological states. It could be observed that almost all types of samples exhibited a higher positive rate and clinical consistence with NST, especially abscess (PPA = 95.7%, NPA = 100%) and CSF (PPA = 88.2%, NPA = 95.7%). In addition, test accuracies of plasma samples (PPA = 77.6%, NPA = 66.7%) were also much better than previously reported (Hu et al., 2021) (**Table 3**). However, statistical performances in SCE samples exhibited a volatile 95% CI, and the clinical consistency of NST was no better than that of culture method (**Supplementary Table S3**), neither in positive nor negative tests. That possibly correlated with the low sample size and low pathogen load in SCE. For samples collected from microbial-colonized sites, NST exhibited 100% PPA but 14.3%–50.0% NPA. This may be due to the fact that, in real-world clinical scenarios, there is a very low probability that no microbes are detected (true negative samples) from the samples collected from microbial-colonized sites, in suspected infectious patients.

We further analyzed the distribution and clinical relevance of detected microorganisms in different body sites. There were more varieties of viral and fewer varieties of fungal pathogens detected from the aseptic body sites, compared with respiratory and urinary tracts (**Figures 4**, **5**). As for clinically relevant bacteria, the top 3 frequently detected species were *Klebsiella pneumoniae*, *E. coli* and *Staphylococcus aureus*, from sterile body sites, while *Mycobacterium tuberculosis*, *E. coli* and *Pseudomonas aeruginosa*, from microbial-colonized sites. These species were in line with the results of several previous studies that investigated pathogens detected from plasma (Eichenberger et al., 2022), SCE (Liu et al., 2023), abscess (Zhao et al., 2021) and respiratory tract (Jin et al., 2022). Among the above species, there was a high concordance in detection of *E. coli*, *P. aeruginosa* and *S. aureus* between NST and culture. Therefore, for common clinical pathogens, it was enough to detect them through gold-standard culture, but it should be mentioned that NST has a faster diagnosis of pathogens than culture (Gu et al., 2021). As for the number of pathogen-derived reads, we recalculated the number of detected reads in each sample, and the top 10 identified pathogens were shown in **Supplementary Figure S1B**. Generally, the number of reads in bacterial pathogens could be considerably higher than that in viral pathogens. For bacterial pathogen which could be detected from both sterile and microbial-colonized body sites, the number of reads detected from the latter sites was greater. Interestingly, the number of detected reads in *K. pneumoniae* in samples collected from sterile body sites could reach 4693.6 ± 1895.0, which was greater than that from the microbial-colonized sites. The varied distribution of the numbers of pathogen-derived reads suggests the complexity of infectious diseases in the real-world clinical scenario, and it is of vital importance with clinical adjudication of NST detection based on the existing technical level.

On the other hand, there are also a number of pathogens better detected with NST than culture, such as *Mycobacterium tuberculosis*, human betaherpesvirus, *Torque teno virus*, and *Pneumocystis jirovecii*. It has been reported that NST can detect fastidious or unculturable microbes (Jin et al., 2022), identify complex infections (Duan et al., 2021), and assist diagnosis of conditional pathogens (Wang et al., 2012). Our study demonstrated that NST performed better than culture test in the detection of fastidious pathogens such as *Mycobacterium* spp., with additional detection of nineteen cases of MTB and six cases of NTM in culture-negative samples. In addition, common clinical conditioning pathogens, including *Candida* (Wang et al., 2012), *Klebsiella pneumoniae*(Chen et al., 2023a), *Pneumocystis jiroveci* (Wang et al., 2022), *Epstein-Barr virus*, and *Adenovirus* were also identified with NST in this study, which were further determined as pathogenic bacteria in combination with clinical symptoms, signs and hemograms of patients. Thus, NST can assist in the precisely and early diagnosis of fastidious and conditional pathogens and serve as an essential supplementary test for the gold standard test. Meanwhile, it should be noticed that NST-detected microbes could be pathogenic or colonized. Therefore, whether the detected microbes are clinically relevant still requires clinical identification and decision making by clinicians.

In addition to the identification of microorganisms, the predictive performance of NST in drug resistance was also notable in this study, which was only reported by a few previous publications (Serpa et al., 2022; Chen et al., 2023b; Xiao et al., 2023). In this study, among 56 microbial strains positive with AMR genes detected by NST only 33 of them were successfully cultured and detected by AST, as shown in **Figure 5B**. There were 58.9% (33 of 56) microbial strains failed to be cultured but were adjudicated as pathogens by clinicians. It should be noted that NST showed a higher detection rate of positive AMR genotype compared with the culture method, which could be actual drug resistance or positive genotype but negative phenotype. Also, it would be a pity that those hypotheses could not be testified because of the negative culture in this study. The failure of gold-standard culture would contribute to the unconscious of neither microbial identification nor drug resistance phenotype. From this perspective, AMR prediction by NST could provide an alternative strategy to infer antimicrobial susceptibility for clinicians and, thus, provide a reference for the selection of clinical treatment scheme, especially for patients with negative culture results. Further study could design prospective experiments for consistency comparisons between AMR genotype and drug resistance phenotype.

This study has the following limitations. First, due to the competitive enrollment of samples, the number of each type of clinical fluid was not consistent, ranging from 23 to 109. Second, the gold-standard culture was used for clinical routine tests of bacterial and fungal pathogens but with difficult application for clinical virus detection. Moreover, this study mainly took microorganisms inherited with DNA into coverage, so RNA virus was not detected or analyzed in this study. Third, the positive rate of culture was relatively low, and the AMR genotype prediction failed to be testified in this study. Further observational studies with large sample size are still necessary to provide more evidence for exploring the diagnostic performance of NST in the clinical setting.

## 5 Conclusions

In conclusion, this study includes seven types of infectious fluid, from 10 clinical centers, and provides evidence for application of NST in real-world clinical scenarios. For samples collected from microbial-colonized sites, NST exhibits more varieties of detected species compared with culture, which needs to be clinically adjudicated. For originally sterile sites, excellent diagnostic performances of NST could support evidence for pathogen identification and antimicrobial treatment. Moreover, NST is potentially valuable in the diagnosis and management of fastidious pathogens, complex infections, and opportunistic pathogen infections, and it is also capable of providing a new strategy to predict antimicrobial susceptibility for clinicians. It should be believed that NST is a promising supplement for gold-standard culture in real-world clinical scenarios.

## Data availability statement

The datasets of Nanopore sequencing for this study have been deposited into CNGB Sequence Archive (CNSA) of China National GeneBank DataBase (CNGBdb) with accession number CNP0004703.

## Ethics statement

The studies involving humans were approved by Ethics Committee of The First Affiliated Hospital of Soochow University. The studies were conducted in accordance with the local legislation and institutional requirements. The participants provided their written informed consent to participate in this study.

## Author contributions

MZ: Data curation, Formal analysis, Project administration, Supervision, Visualization, Writing – original draft. YYZ: Project administration, Supervision, Writing – original draft. LC: Data curation, Formal analysis, Visualization, Writing – original draft. XY: Investigation, Methodology, Writing – original draft. TX: Investigation, Methodology, Writing – original draft. MF: Investigation, Methodology, Writing – original draft. YH: Validation, Writing – original draft. YZ: Validation, Writing – original draft. BZ: Validation, Writing – original draft. JC: Validation, Writing – original draft. JL: Project administration, Supervision, Writing – review & editing. DS: Funding acquisition, Project administration, Supervision, Writing – review & editing. SL: Conceptualization, Data curation, Formal analysis, Visualization, Writing – review & editing. CZ: Conceptualization, Writing – review & editing. WZ: Conceptualization, Funding acquisition, Resources, Writing – review & editing.
