# Supplementary material for: Nanopore sequencing of infectious fluid is a promising supplement for gold-standard culture in real-world clinical scenario
Source: Front Cell Infect Microbiol. 2024 Jan 30;14:1330788. doi: 10.3389/fcimb.2024.1330788 (PMC10861792; doi:10.3389/fcimb.2024.1330788)
Supplement: Supplementary file 1 [file DataSheet_1.pdf]

Supplementary Material

1 Supplementary Data

Not applicable.

2 Supplementary Figures and Tables

2.1 Supplementary Figures

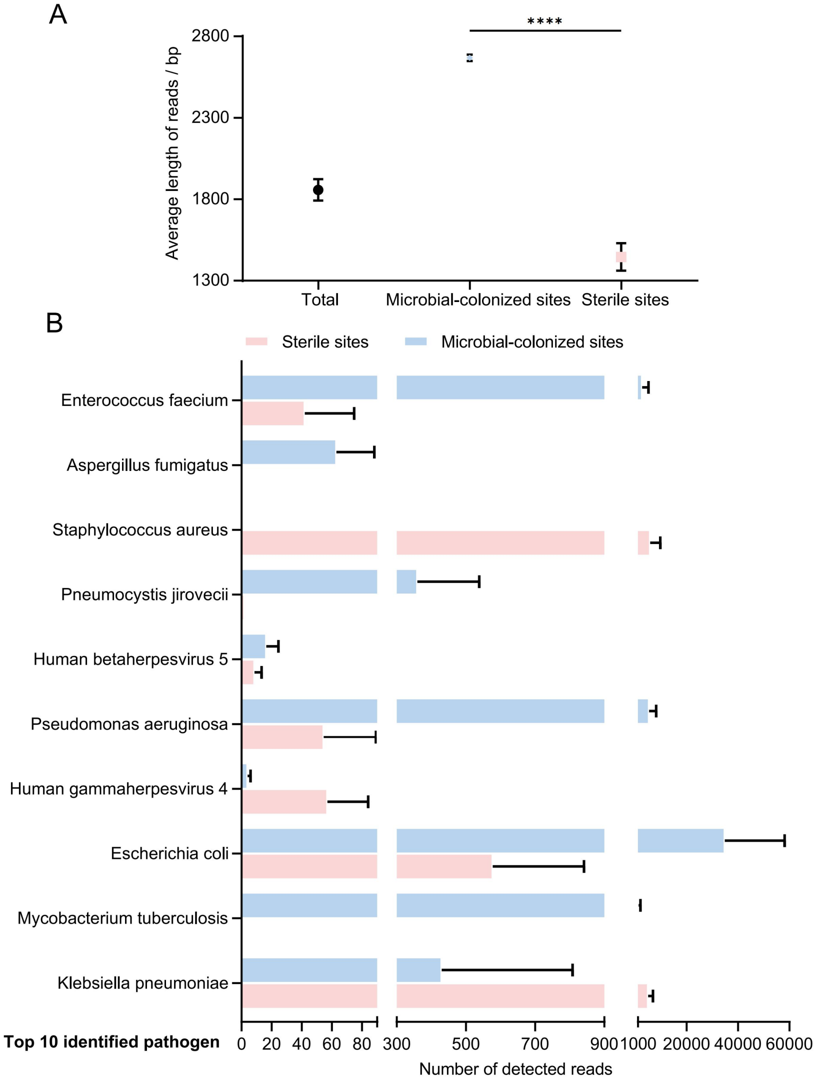

Supplementary Figure S1. Basic sequencing results of NST detected reads. (a). Average length of reads detected from each sample. Total, total samples (n = 297). Microbial-colonized sites (n = 100). Sterile sites (n = 197). **(b).** Number of detected reads of top 10 identified pathogen in each sample. Values were shown as Mean ± SEM. ****p < 0.0001.

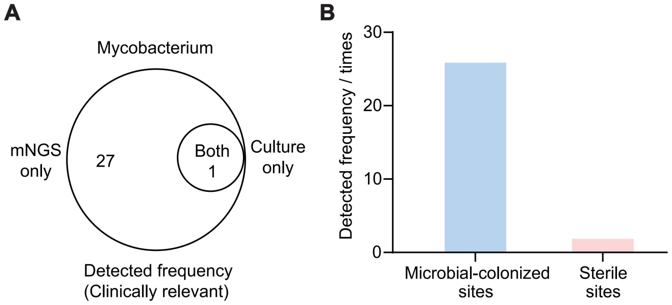

Supplementary Figure S2. Detective distribution of *Mycobacterium*. (a). Detected frequencies of different methodology. **(b).** Detected frequencies of different body sites.

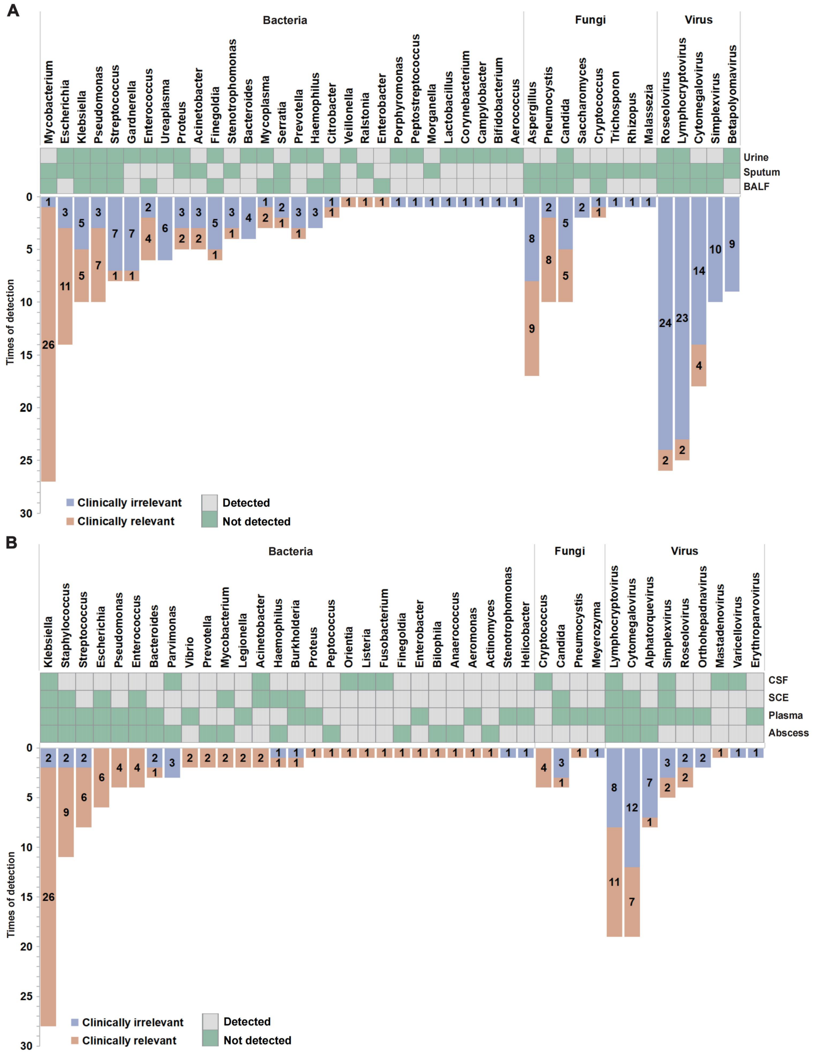

Supplementary Figure S3. Proportions of microorganisms identified with NST (Genus level) and the clinical relevance distribution of each genus. (a). Microorganisms detected from body sites with colonized microbiome. **(b).** Microorganisms detected from body sites with non-colonized microbiome. BAL, bronchoalveolar lavage fluid. Spu, Sputum. Uri, urine. Abs, abscess. Pla, plasma. CSF, cerebrospinal fluid. SCE, serous cavity effusion.

2.2 Supplementary Tables

Supplementary Table S1. Inclusion and exclusion criteria of this study.

Criteria	Details
Inclusion criteria	(1) Patients over 18 years old;<br>(2) Patients exhibited typical clinical signs of infection, such as fever for all suspected infections, cough and expectoration for pulmonary infection, localized abscess formation, headache or lose consciousness for central nervous system infection, chest tightness or abdominal bloating for thoracic or abdominal infection;<br>(3) Patients whose diagnosis of infections supported by radiological evidence or routine laboratory tests, such as abnormal Procalcitonin (over 0.5 ng/mL) for bloodstream infection, elevated C-Reactive Protein (over 4.0 mg/L) for pulmonary infection, and positive detection of urine white blood cells for urinary tract infection.
Exclusion criteria	(1) Patients with contraindications to cerebrospinal fluid or thoracic and abdominal fluid puncture<br>(2) Patients refusing to sign the consent documents or collect the medical records<br>(3) Patients who were lost to follow-up

Supplementary Table S2. Sample characteristics of enrolled patients.

Sample characteristics (n = 297)	Value
**Sample type, n (%)**	
Abscess	24 (8.1%)
BALF	40 (13.5%)
CSF	40 (13.5%)
Plasma	109 (36.7%)
SCE	24 (8.1%)
Sputum	36 (12.1%)
Urine	24 (8.1%)
**Organism cultured, n (%)**	
Enterobacteriaceae	33 (10.9%)
Staphylococcus	12 (4%)
Pseudomonas aeruginosa	7 (2.3%)
Streptococcus	5 (1.7%)
Acinetobacter	5 (1.7%)

Sample characteristics (n = 297)	Value
Stenotrophomonas maltophilia	2 (0.7%)
Mycobacterium tuberculosis	1 (0.3%)
Enterococcus	1 (0.3%)
Burkholderia cepacia	1 (0.3%)
Other bacteria	10 (3.3%)
Fungi	20 (6.6%)
Negative	205 (67.9%)

Supplementary Table S3. Detective result overview of positive and negative tests.

Sample type	Positive test (n)		Positive test clinical consistency (n, %)		Negative test (n)		Negative test clinical consistency (n, %)	
	Cul	NST	Cul	NST	Cul	NST	Cul	NST
BALF	12	35	8 (66.67)	30 (85.71)	22	5	2 (9.09)	1 (20.00)
Sputum	16	34	13 (81.25)	31 (91.18)	16	2	1 (6.25)	1 (50.00)
Urine	13	23	12 (92.31)	17 (73.91)	11	1	1 (9.09)	0 (0)
Abscess	12	22	12 (100)	22 (100)	9	2	1 (11.11)	1 (50.00)
Plasma	30	62	26 (86.67)	45 (72.58)	72	47	20 (27.78)	16 (34.04)
CSF	5	16	5 (100)	15 (93.75)	33	24	13 (39.39)	13 (54.17)
SCE	4	8	4 (100)	5 (62.50)	16	16	3 (18.75)	3 (18.75)
**Total**	92	200	80 (86.96)	165 (82.50)	179	97	41 (22.91)	35 (36.08)

Supplementary Table S4. Statistical performance of different types of samples (n = 271, gold standard).

Sample type	TP	FP	TN	FN	Sensitivity	Specificity	PPV	NPV
BALF	5	23	5	0	100 (46.3-100)	17.9 (6.8-37.6)	17.9 (6.8-37.6)	100 (46.3-100)
Sputum	14	16	2	0	100 (73.2-100)	11.1 (1.9-36.1)	46.7 (28.8-65.4)	100 (19.8-100)
Urine	10	13	1	0	100 (65.5-100)	7.1 (0.4-35.8)	43.5 (23.9-65.1)	100 (5.5-100)
Abscess	12	9	1	0	100 (69.9-100)	10.0 (0.5-45.9)	57.1 (34.4-77.4)	100 (5.5-100)
Plasma	12	48	34	8	60.0 (36.4-80.0)	41.5 (30.9-52.9)	20.0 (11.2-32.7)	81.0 (65.4-90.9)
CSF	3	12	22	1	75.0	64.7	20.0	95.7

Sample type	TP	FP	TN	FN	Sensitivity	Specificity	PPV	NPV
					(21.9-98.7)	(46.5-79.7)	(5.3-48.6)	(76.0-99.8)
SCE	2	4	13	1	66.7<br>(12.5-98.2)	76.5<br>(49.8-92.2)	33.3<br>(6.0-75.9)	92.9<br>(64.2-99.6)
Total	58	125	78	10	85.2<br>(74.2-92.3)	38.4<br>(31.8-45.5)	31.7<br>(25.1-39.0)	88.6<br>(79.7-94.1)

TP, FP, FN and TN were shown as numbers of samples. Sensitivity, Specificity, PPV and NPV were shown as % (95% confidence intervals), and were calculated with *http://vassarstats.net/clin1.html#return*.
